# Independent and synergistic effects of maternal and paternal obesogenic diets on offspring early‐life outcomes in the rat

**DOI:** 10.1113/EP093885

**Published:** 2026-06-12

**Authors:** Khurram Jahangir Toor, Mehwish Abbasi, Swetha Muralidharan, Elisa Weiss, Elwyn C. Firth, Anna P. Ponnampalam, David S. Musson, Mark H. Vickers, Benjamin B. Albert

**Affiliations:** ^1^ Liggins Institute The University of Auckland Auckland New Zealand; ^2^ Department of Nutrition and Dietetics The University of Auckland Auckland New Zealand; ^3^ Department of Physiology, Healthy Hearts for Aotearoa New Zealand – Pūtahi Manawa The University of Auckland Auckland New Zealand

**Keywords:** developmental programming, high fat, high sugar, maternal obesity, obesity, parental obesity, paternal obesity

## Abstract

Independent effects of maternal obesogenic diets on offspring outcomes are acknowledged, but data on paternal and combined maternal–paternal obesity remain limited. We investigated independent and combined maternal–paternal effects of an obesogenic (OB) high‐fat, high‐sugar diet on offspring outcomes. Male (pat) and female (mat) rats were fed a control diet (CON) or OB diet for 5 weeks pre‐mating. Four groups were established: matCON‐patCON, matCON‐patOB, matOB‐patCON and matOB‐patOB. Offspring outcomes included birth weight and naso‐anal length, survival rates to weaning, weaning weight, retroperitoneal fat mass and plasma leptin. Mating success was reduced in OB‐exposed fathers. Male and female offspring from mothers fed the OB diet had lower birth weights. In males, birth weights were further reduced in the matOB‐patOB group. Combined maternal–paternal obesity was also associated with shorter body length and higher neonatal mortality in offspring of both sexes. Postnatal survival was lower with maternal obesity and worsened with combined maternal–paternal obesity. At weaning, male offspring exposed to maternal or combined maternal–paternal obesity showed increased body weight, adiposity and plasma leptin concentrations. In females, body weight at weaning was higher across all parental obesogenic diet groups, and increased adiposity and plasma leptin concentrations were observed in maternal and combined maternal–paternal obesity. Parental obesity had independent, combined and sex‐specific effects on early‐life outcomes, most severe with combined maternal–paternal obesity. These findings emphasise the interacting role of maternal and paternal nutrition in shaping early‐life outcomes and highlight the need for research to determine effective strategies to optimise the health of both parents prior to conception.

## Abstract (highlights)

- **What is the central question of this study?**
  What are the independent and combined effects of maternal and paternal obesogenic diets on early‐life outcomes?
- **What is the main finding and its importance?**
  A maternal obesogenic diet has a stronger negative effect on early‐life outcomes, such as mortality, birth weight and weaning adiposity, but when combined with a paternal obesogenic diet the impact on survival is greater. This research highlights the need to optimise the health of both parents prior to conception, and that there are interacting effects of maternal and paternal health on the offspring.

## 1 INTRODUCTION

Obesity is a chronic global health challenge affecting >1 billion people, including ∼880 million adults and 159 million children and adolescents (NCD Risk Factor Collaboration, 2024). Obesity is a major contributor to non‐communicable diseases (NCDs), such as type 2 diabetes, cardiovascular disease, stroke and cancer, and is responsible for one in eight NCD‐related deaths worldwide (NCD Risk Factor Collaboration, 2024; World Obesity Federation, 2025). The burden is shifting rapidly to low‐ and middle‐income countries, where it is expected that 79% of adults and 88% of children will be affected by obesity by 2035 (World Obesity Federation, 2024). The economic impact of obesity is forecast to reach $4.32 trillion annually by 2035, equivalent to nearly 3% of global Gross Doestic Product, a level comparable to the cost of the COVID‐19 pandemic (World Obesity Federation, 2023).

Maternal obesity affects approximately one in six pregnancies globally, and together with overweight status, affects nearly 44% of all pregnancies worldwide (Kalantari et al., 2024; Kent et al., 2024). Maternal obesity in humans is linked to increased risk of congenital anomalies and metabolic disorders in offspring (Kweon et al., 2024; Stothard et al., 2009), and in animal (mainly rodent) models, maternal obesogenic diets impair adipose tissue development, increase postnatal fat accumulation and alter mitochondrial function (Mendonça et al., 2025; Xhonneux et al., 2023). Meta‐analysis of preclinical studies confirmed that maternal high‐fat diet exposure increased offspring adiposity, insulin resistance and lipid dysregulation (Ribaroff et al., 2017). Despite extensive maternal‐focused research, most studies have examined maternal obesity in isolation, leaving the impact of combined maternal and paternal obesogenic diet on offspring development underexplored.

Paternal obesity is a growing concern in developmental research but is underrepresented in comparison to maternal studies (Xie, 2024). In human studies, elevated body mass index (BMI) is associated with abnormal sperm morphology, indicating poorer sperm quality (Darand et al., 2023; Elbardisi et al., 2025), and epidemiological data also link paternal obesity with adverse cardiometabolic outcomes in offspring, including increased BMI, insulin resistance and elevated blood pressure (Zhang et al., 2024). In rodent models, paternal exposure to obesogenic diets may induce epigenetic modifications in sperm, such as altered DNA methylation and changes in non‐coding RNA expression, which could influence gene expression in the developing embryo (Akhatova et al., 2025; Haberman et al., 2024). Emerging evidence suggests that these paternal diet‐induced epigenetic changes might contribute to offspring metabolic disturbances, including impaired insulin sensitivity and cardiovascular dysfunction (Ng et al., 2010; Noor et al., 2019; Sivakumar et al., 2024; Tian et al., 2025). However, previous studies of paternal obesity have focused on metabolic phenotypes later in life, and no studies to our knowledge have evaluated whether paternal obesity influences early postnatal outcomes, such as neonatal mortality and early‐life survival. Moreover, the interactions between maternal and paternal obesity on early‐life outcomes and maternal outcomes after pregnancy have also not been examined.

Human cohort studies suggest that when both mothers and fathers have obesity there is an amplified risk for higher BMI, metabolic syndrome and insulin resistance in their offspring (Fuemmeler et al., 2013; Jurado‐Sumariva et al., 2025; Yang et al., 2020; Zalbahar et al., 2017). However, such observational studies have limitations, most importantly in the difficulty of controlling for the many environmental and social confounding factors that might link the health of mothers, fathers and their children, including diet and physical activity, and the way the parents provide care to their children. Furthermore, studies examining associations between paternal factors, such as BMI, and the outcomes of children often do not have measures of the paternal factors prior to conception, which is the time point of relevance if the health of the father is to affect the development and physiology of the child directly. In contrast, experimental animal models permit precise control of the parental environment from preconception, such that interventional groups differ solely by the intervention or interventions of interest. This allows cause to be attributed to the intervention.

Studies in the mouse confirm additive adverse effects of combined maternal–paternal obesity on embryo development, placental function and offspring metabolic health (Finger et al., 2015; McPherson et al., 2015; Ornellas et al., 2015). Despite this growing evidence, most studies continue to focus on maternal obesity alone, leaving the independent and combined maternal–paternal effects of obesogenic diets insufficiently resolved.

In this study, we examine the effects of maternal, paternal and combined maternal–paternal obesogenic diets on early‐life outcomes using a balanced 2 × 2 factorial design. By evaluating reproductive success, neonatal mortality, postnatal survival, early growth trajectories and adiposity across all parental diet combinations, we aimed to quantify the effects of maternal and paternal obesity and interaction effects across this early‐life period.

## 2 MATERIALS AND METHODS

### 2.1 Ethics statement

All experimental procedures were approved by the University of Auckland Animal Ethics Committee (approval #R0126450) and complied with the New Zealand Animal Welfare Act (1999) and the ethical principles under which *Experimental Physiology* operates.

### 2.2 Animals

Female (*n =* 100) and male (*n =* 40) Sprague–Dawley rats were sourced from the Vernon Jenson Unit (University of Auckland) at an early post‐weaning age. A smaller number of male rats were used in this study, because males could be mated more than once. Of the 100 females, ∼80 were planned to become pregnant, whereas ∼20 were to undergo assessments without mating.

Rats were housed in groups of four or five per cage in standard housing conditions: 12 h–12 h light–dark cycle (lights on at 06.00 h), 22°C ± 1°C, and ad libitum access to a standard chow diet (Teklad 2018 Rodent Diet) and water. Body weights and food consumption were monitored every 6 days until day 65. Food intake was measured at the cage level, but expressed as per animal intake by dividing by the number of animals (four or five) and the current mean body weight of animals in the cage.

### 2.3 Diet

On day 65, half of the female and male rats were randomly allocated to receive a high‐fat, high‐sucrose obesogenic (OB) diet (SF23‐120 Specialty Feeds, WA, Australia) to induce obesity and metabolic dysfunction. The remaining female and male rats continued on the control (CON) chow diet. From day 65, body weight and food intake were monitored every 3 days. Table 1 shows the macronutrient composition and major ingredients of the OB and CON diets. A high‐fat, high‐sucrose diet has been demonstrated to induce obesity, insulin resistance and ectopic fat deposition in Sprague–Dawley rats (Zhou et al., 2014).

**TABLE 1:** Macronutrient composition of the CON (chow) and OB (high‐fat high‐sucrose) diets.

Parameter	CON diet, Teklad 2018 rodent diet	OB diet, SF23‐120 specialty feeds
Energy density, kcal/g	3.1	4.42
Macronutrient composition, % weight		
Protein	18.4	17.5
Fat	6.0	21.0
Total saturated	0.9	13.7
Total monounsaturated	1.3	5.9
Total polyunsaturated	3.4	0.6
Digestible carbohydrate	44.2	47.5
Sucrose^a^	–	34.1
Crude fibre	3.8	4.7
Energy contribution, % energy		
Protein	24	16
Fat	18	41
Total carbohydrate	58	43
Sucrose^a^	–	31
Major ingredients	Ground wheat, ground corn, wheat middlings, dehulled soybean meal, corn gluten meal, soybean oil, calcium carbonate, dicalcium phosphate, brewers dried yeast, iodized salt	Lactic acid casein (195 g/kg), sucrose (341 g/kg), clarified butter (210 g/kg), cellulose (50 g/kg) wheat starch (22 g/kg), dextrinised starch (132 g/kg), calcium carbonate (17.1 g/kg), sodium chloride (2.6 g/kg)

^a^ The CON diet had no added sucrose. Although the manufacturer does not report the sucrose content explicitly, the sucrose content of wheat and corn is very low. Ingredients by weight were provided by the manufacturer of the OB but not the CON diet.

### 2.4 Mating

**FIGURE 1:**
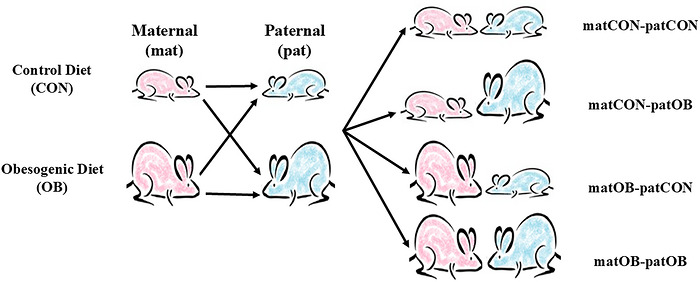
Experimental design for assessing the impact of maternal and paternal diets on offspring outcomes. Female and male rats were assigned to either the Control (CON) or Obesogenic (OB) diet. After 5 weeks on the respective diets, mating was conducted to generate four parental diet groups based on the maternal (mat) and paternal (pat) diet: matCON‐patCON, matCON‐patOB, matOB‐patCON and matOB‐patOB. As an example, matCON‐patOB indicates a parental diet group where the mother consumed a control diet and the father consumed an obesogenic diet.

From 100 days of age, female rats were time mated using an oestrous cycle monitor (Model MK11, Muromachi, Japan). Day 1 of pregnancy was determined by detection of spermatozoa following vaginal lavage. Males and females were matched to produce equal numbers of matings for each of four combinatorial groups (matCON‐patCON, matCON‐patOB, matOB‐patCON and matOB‐patOB; Figure 1). Each male was paired with females from both dietary groups to reduce genetic bias across offspring groups. Males were given at least two mating opportunities. Successful mating was confirmed by vaginal lavage and identification of sperm under light microscopy. Those males that did not result in a positive vaginal lavage after two mating attempts were excluded from further mating. Following confirmation of pregnancy, females were housed individually and fed the same diet as consumed prior to mating, which was maintained throughout the lactation period; body weight and food intake of the pregnant females were recorded every 3 days.

### 2.5 Phenotyping of parent animals

To examine the effect of diet on parental body composition and metabolism, fathers and a subset of unmated females were assessed after the completion of mating. Between days 125 and 150, males (*n =* 20) and unmated females (*n =* 18) underwent isoflurane anaesthesia to assess body composition using dual‐energy X‐ray absorptiometry (Lunar Prodigy 2000, General Electric, Madison, WI, USA). Following a 6 h fast, animals were euthanised by pentobarbitone sodium anaesthesia (60 mg/kg; intraperitoneal) followed by decapitation. Trunk blood was collected into heparinised tubes and stored on ice until centrifugation and removal of plasma for analysis. The glucose concentration was measured by point‐of‐care glucometer (Abbott Freestyle, NZ) and plasma insulin concentration was measured using a rat‐specific ultra‐sensitive ELISA kit (Crystal Chem, IL, USA; catalogue No. 90060) with an intra‐assay coefficient of variation of 3.7% and inter‐assay coefficient of variation of 13.7%. HOMA‐IR (homeostatic model assessment for insulin resistance) and HOMA‐β (homeostatic model assessment for beta‐cell function) were calculated (Matthews et al., 1985). Liver and retroperitoneal fat were weighed and represented as a percentage of body weight (%BW).

### 2.6 Offspring assessments

Following delivery, all pups were weighed, sexed, and the nose‐to‐anus (NA) length measured. Dead pups were sexed and recorded.

On postnatal day 2 (PN2), pups were weighed and sexed. To standardise pup nutrition, litter size was adjusted to eight pups per litter, where possible four male and four female. Unused pups were killed by decapitation. Pups that appeared unwell were preferentially selected for removal. Litters were kept with their mothers until weaning at day 21 (PN21). Maternal body weight and food consumption were measured, and pups were sexed and weighed every 3 days. Whenever offspring were assessed, including as neonates, the period of maternal separation was kept <2 min, and the dam remained within sight and sound throughout the procedure. Neonatal mortality and postnatal survival to weaning were recorded.

On PN21, following a 6 h fast, the pups and dams were anaesthetised using pentobarbitone sodium injection (60 mg/kg; intraperitoneal), followed by decapitation. Blood was collected in heparinised tubes. Leptin concentrations in plasma collected from PN21 pups were measured by rat‐specific ELISA (Crystal Chem, IL, USA; catalogue No. 90040) with intra‐assay coefficient of variation of 5.4% and inter‐assay coefficient of variation of 13.0%. Liver and retroperitoneal fat pads were completely excised and weighed.

### 2.7 Statistical analysis

All data were analysed using GraphPad Prism v.10.3.1, with statistical significance set at *P* < 0.05. Data normality was assessed using the Shapiro–Wilk test to inform the selection of an appropriate test. For comparisons of metabolic parameters (e.g., HOMA‐IR, HOMA‐β, insulin and glucose) between parent obesogenic diet groups versus controls, Student's unpaired *t*‐tests were performed. In the analysis of fertility outcomes, only the first two mating attempts were considered, because subsequent attempts were allowed only to successful males. Mating success was analysed using Fisher's exact test. To assess parental outcomes, two‐way repeated‐measures ANOVA was used to compare weight curves and caloric intake (normalised to body weight) between control and obesogenic diet groups in males and females. Dam outcomes were analysed using two‐way ANOVA to assess the main effects of maternal diet, paternal diet and their interaction, followed by Tukey's HSD *post hoc* tests. Data are presented as the mean ± SD. For preconception food intake measurements obtained during group housing, the cage was considered the experimental unit.

Offspring outcomes (e.g., birth weight, postnatal body length, retroperitoneal fat mass as %BW, and plasma leptin at weaning) were analysed using two‐way ANOVA to assess the effects of maternal diet, paternal diet and their interaction, with Tukey's HSD for *post hoc* comparisons. Survival outcomes from PN2 to PN21 were evaluated using log‐rank (Mantel–Cox) tests to compare survival curves across diet groups. Neonatal mortality was analysed using Fisher's exact test. *z*‐Scores of offspring body weight were calculated at PN1 and PN21 using the matCON‐patCON group as reference. Catch‐up growth was assessed as Δ*z*‐score (PN21 − PN1). Given that pups could not be identified individually over time, the Δ*z*‐score (PN21 − PN1) is based on mean group *z*‐scores, and no measures of variation or significance could be calculated. Analysis was performed separately for male and female offspring, with the exception of neonatal mortality and survival to weaning.

## 3 RESULTS

### 3.1 Effect of the obesogenic diet on parental body weight, body composition and metabolic profiles

**FIGURE 2:**
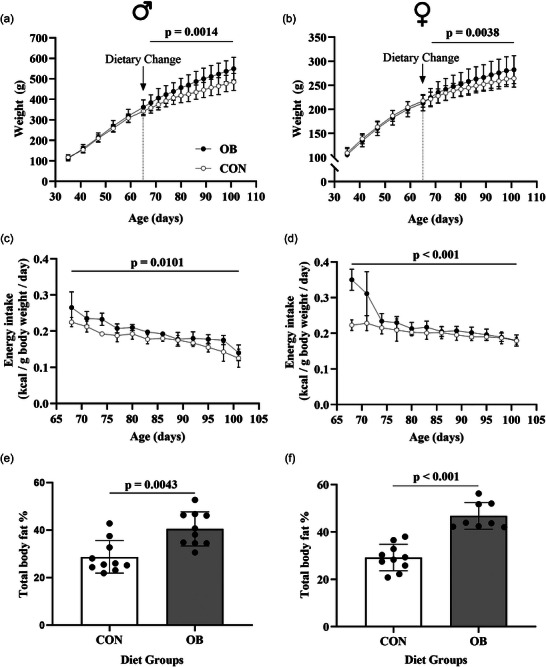
Preconception body weight, energy intake and body fat percentage in male and female rats exposed to CON or OB diets. (a) Body weight in males (*n =* 40). (b) Body weight in females (*n =* 100). (c) Energy intake in males. (d) Energy intake in females. (e) Percentage body fat measured by dual‐energy X‐ray absorptiometry in males (*n =* 20). (f) Percentage body fat in females (*n =* 18). Body weight and total body fat percentage are based on individual animals, whereas energy intake was determined at the cage level (males: 4 CON and 4 OB cages; females: 11 CON and 10 OB cages). Data are presented as the mean ± SD.

Male rats fed the OB diet had higher body weight and energy intake compared with those fed the CON diet (Figure 2; Table 2). They also had greater total fat mass, lower lean mass, leading to greater total body fat percentage, and had higher bone mineral content and bone mineral density. There were no differences in relative liver weights (%BW). Fasting plasma insulin concentrations, HOMA‐IR and HOMA‐β indices were also increased in male rats fed the OB diet compared with control males, indicative of reduced insulin sensitivity. There were no differences in fasting plasma glucose.

**TABLE 2:** Metabolic and body composition outcomes in parental rats following exposure to CON or OB diets.

	Parameter	Female (unmated)			Male parent		
		matCON	matOB	*P*‐value	patCON	patOB	*P*‐value
	*n*	52	48		20	20	
Premating weight	Body weight, g	265.4 ± 18.5	282.2 ± 28.7	**<0.001**	484.7 ± 40.8	549.1 ± 55.3	**<0.001**
Final measurements of male parents and unmated females	*n*	10	8		10	10	
	Absolute body fat, g	77.2 ± 17.7	141.9 ± 20.4	**<0.001**	153.5 ± 43.5	231.2 ± 50.4	**0.0017**
	Absolute lean mass, g	186.4 ± 14.2	161.6 ± 20.7	**0.0081**	377.3 ± 44.7	337.1 ± 38.9	**0.0457**
	Percentage body fat	29.2 ± 5.6	46.7 ± 5.6	**<0.001**	28.7 ± 6.8	40.5 ± 7.2	**0.0014**
	Percentage lean mass	70.8 ± 5.5	53.3 ± 5.7	**<0.001**	71.3 ± 6.8	59.5 ± 7.1	**0.0014**
	Fat to lean mass ratio	0.419 ± 0.114	0.898 ± 0.213	**<0.001**	0.416 ± 0.151	0.704 ± 0.215	**0.0027**
	Bone mineral content, g	9.01 ± 0.56	11.03 ± 0.95	**<0.001**	15.33 ± 1.38	17.34 ± 1.35	**0.0040**
	Bone mineral density, g/cm^2^	0.1597 ± 0.0038	0.1683 ± 0.0062	**0.0024**	0.1812 ± 0.0048	0.1887 ± 0.0057	**0.0053**
	Liver, %BW	2.89 ± 0.16	2.74 ± 0.33	0.219	2.60 ± 0.36	2.74 ± 0.18	0.290
	Retroperitoneal fat, %BW	0.73 ± 0.16	1.61 ± 0.42	**<0.001**	1.05 ± 0.41	1.99 ± 0.73	**0.0023**
Glucose homeostasis	Fasting glucose, mmol/L	8.38 ± 0.78	9.55 ± 0.88	**0.0089**	7.99 ± 1.01	8.64 ± 1.68	0.312
	Fasting insulin, ng/mL	4.60 ± 1.96	6.02 ± 2.17	0.165	2.64 ± 0.87	5.32 ± 2.95	**0.013**
	HOMA‐IR	41.56 ± 18.91	62.23 ± 26.72	0.0724	22.93 ± 9.31	50.76 ± 35.46	**0.0274**
	HOMA‐β	453.42 ± 184.69	475.45 ± 144.89	0.786	286.22 ± 88.94	524.85 ± 244.75	**0.0096**

*Note*: Premating weights were measured at day 100. Final measurements were taken following the completion of all mating in a subset of animals (female: Control diet (CON) 10, Obesogenic diet (OB) 8; male: CON 10, OB 10). Females that underwent final measurements had not undergone mating, but followed the same dietary manipulation as mated females. Data are expressed as the mean ± SD. P‐values <0.05 are indicated in bold.

Abbreviations: %BW, percentage of body weight; matCON, female animals from the parent generation that consumed a control diet; matOB, female animals from the parent generation that consumed an obesogenic diet; patCON, male animals from the parent generation that consumed a control diet; patOB, male animals from the parent generation that consumed an obesogenic diet.

Likewise, among female rats fed the OB diet, there was an increase in body weight and energy intake, and differences in body composition, including greater total body fat, lower lean mass, greater body fat percentage, greater bone mineral content and bone mineral density compared with those fed the CON diet (Figure 2; Table 2). There were no differences in relative liver weight (%BW). Fasting plasma glucose concentrations were significantly higher in female parents fed the OB diet. Although there was no difference in fasting plasma insulin, there was a trend to higher HOMA‐IR in females fed the OB diet compared with females consuming the CON diet (*P* = 0.0724).

### 3.2 Mating success across parental diet groups

**FIGURE 3:**
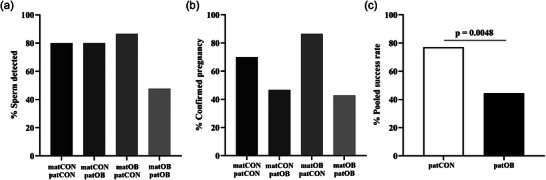
Mating success in male rats across parental diet groups. (a) Success based on sperm detection following vaginal lavage. (b) Success based on confirmed pregnancy. (c) Pooled success rates to isolate the paternal diet effect on confirmed pregnancy. For this comparison, patCON values combine matCON‐patCON and matOB‐patCON, whereas patOB values combine matCON‐patOB and matOB‐patOB. Diet groups: matCON‐patCON (*n =* 20), matCON‐patOB (*n =* 15), matOB‐patCON (*n =* 15) and matOB‐patOB (*n =* 21). Data are expressed as percentages. Statistical differences were assessed by Fisher's exact test (a, *P* = 0.0466; and b, *P* = 0.0293, for an overall difference between groups).

Mating success across parental diet groups was evaluated using two criteria: sperm detection following vaginal lavage (Figure 3a) and confirmed pregnancy (where there was subsequent rapid weight gain and delivery of a litter; Figure 3b). For both sperm detection and confirmed pregnancy, success rates differed significantly among the four parental diet groups. Sperm detection was lowest in the matOB‐patOB group, and confirmed pregnancy was lowest in both the matCON‐patOB and the matOB‐patOB groups. A pooled analysis based on confirmed pregnancies showed significantly lower success rates in patOB compared with patCON (Figure 3c).

### 3.3 Dam characteristics during pregnancy and lactation

**FIGURE 4:**
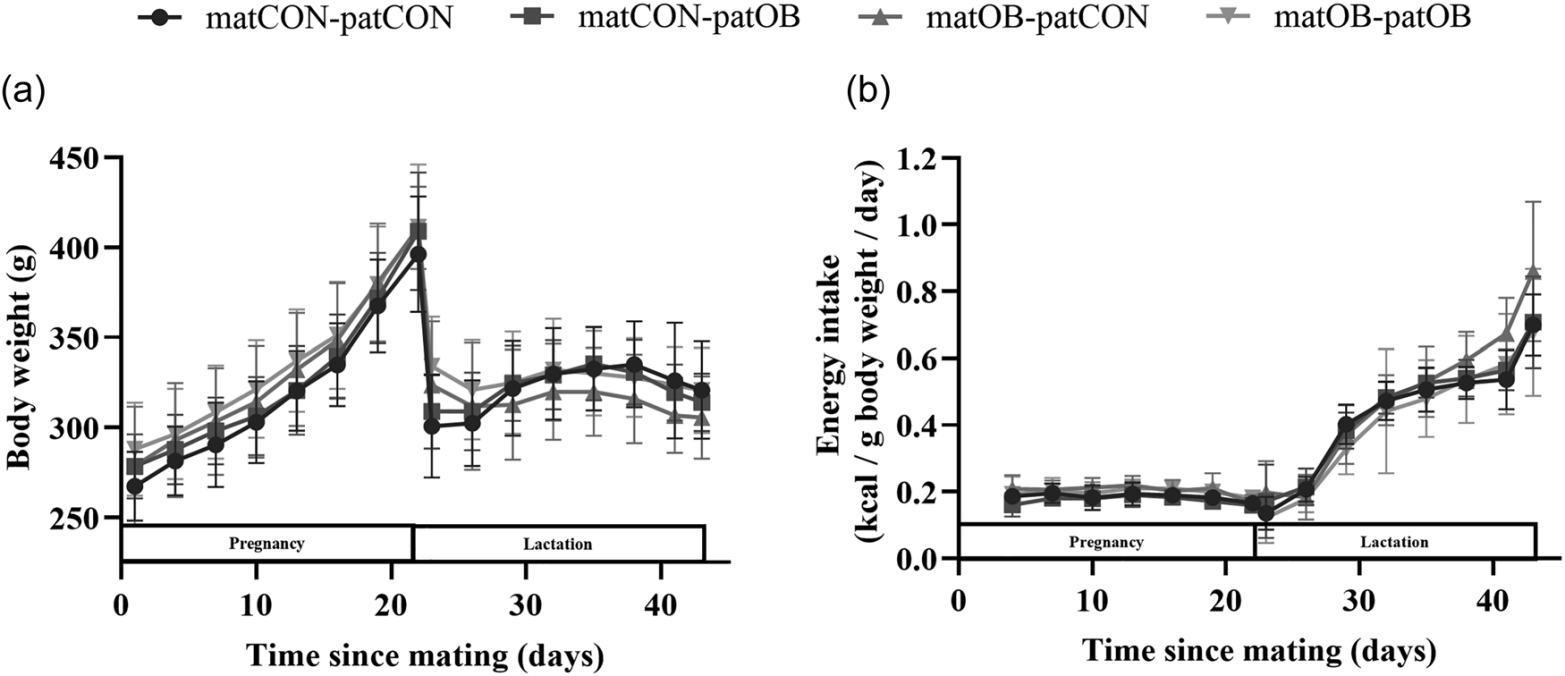
Maternal weight trajectory and maternal energy consumption across four diet groups. (a) Maternal body weight trajectory throughout gestation and lactation across the four diet combinations. (b) Maternal energy intake during pregnancy and lactation, measured in individually housed dams and expressed per animal. Diet groups: matCON‐patCON (*n =* 15), matCON‐patOB (*n =* 16), matOB‐patCON (*n =* 18) and matOB‐patOB (*n =* 15). Data are presented as the mean ± SD.

Dam characteristics during pregnancy and lactation are presented in Table 3, and weight and food intake trajectories are shown in Figure 4. Overall, a maternal obesogenic diet was associated with greater caloric intake during pregnancy, greater weight on postpartum day 1, greater weight loss across the lactation period and increased retroperitoneal body fat (%BW) and liver weight (%BW) at the end of lactation. In contrast, a paternal obesogenic diet was associated with lower gestational weight gain in dams, but also greater retroperitoneal body fat (%BW) in dams at the end of lactation.

**TABLE 3:** Dams body weight, food intake and metabolic markers across pregnancy and lactation.

Parameter	matCON‐patCON	matCON‐ patOB	matOB‐ patCON	matOB‐ patOB	Maternal diet effect	Paternal diet effect	Parental diet interaction
*n*	15	16	18	15			
Gestational day 1 weight, g	267.20 ± 19.21	278.31 ± 17.92	278.47 ± 33.01	287.92 ± 25.87	0.114	0.120	0.899
Gestational day 19 weight, g	367.40 ± 25.66	371.75 ± 25.08	380.33 ± 32.87	379.25 ± 32.35	0.187	0.832	0.724
Gestational weight change, g	100.20 ± 13.57	93.44 ± 15.22	101.87 ± 14.17	91.33 ± 16.77	0.956	**0.0321**	0.634
Pregnancy cumulative food intake, kcal	1070.5 ± 120.2	1025.7 ± 110.8	1240.3 ± 160.0	1205.9 ± 132.8	**<0.001**	0.547	0.344
Postpartum day 1 weight, g	300.60 ± 28.65^a^	308.94 ± 20.63^ab^	323.56 ± 35.31^ab^	334.08 ± 27.44^b^	**0.0038**	0.123	0.675
Postpartum day 21 weight, g	320.73 ± 26.92	314.00 ± 16.02	305.44 ± 23.00	320.58 ± 23.62	0.387	0.402	0.0534
Weight change across lactation period, g	20.13 ± 17.11^a^	5.06 ± 15.28^a^	−15.80 ± 25.65^b^	−13.50 ± 18.90^b^	**<0.001**	0.228	0.0992
Lactation cumulative food intake, kcal	3038.4 ± 169.9	3095.2 ± 208.0	3145.2 ± 330.8	2905.4 ± 620.8	0.661	0.334	0.120
Maternal fasting glucose, mmol/L	7.21 ± 1.48	7.53 ± 1.37	7.49 ± 1.46	7.94 ± 1.69	0.380	0.330	0.863
Liver, %BW	3.59 ± 0.21^a^	3.62 ± 0.17^a^	4.92 ± 0.73^b^	4.46 ± 1.24^b^	**<0.001**	0.239	0.185
Retroperitoneal fat, %BW	0.34 ± 0.13^a^	0.41 ± 0.15^a^	0.48 ± 0.29^ab^	0.66 ± 0.27^b^	**0.0012**	**0.0357**	0.327

*Note*: Dam characteristics across four diet groups. Outcomes include gestational and postpartum weights, weight changes, food intake, fasting glucose, liver (%BW) and retroperitoneal fat (%BW). Data are presented as the mean ± SD. Groups that do not share a superscript letter in common are significantly different (*P* < 0.05; shown in bold) by parametric two‐way ANOVA, with Tukey's multiple comparison test.

Abbreviations: %BW, percentage of body weight; CON, control diet, OB, obesogenic diet; matCON‐patCON, the group where the mother and father consumed a control diet; matCON‐patOB, the group where the mother consumed a control diet diet and the father consumed an obesogenic diet; matOB‐patCON, the group where the mother consumed an obesogenic diet and the father consumed a control diet; matOB‐patOB, the group where the mother and father consumed an obesogenic diet.

Although there were no between‐group differences for fasting glucose in dams at the end of lactation, the maternal obesogenic diet was associated with higher relative liver weight (%BW). *Post hoc* testing indicated that both maternal obesogenic diet groups (matOB‐patCON and matOB‐patOB) had greater relative liver weight than the maternal control groups. Both the maternal and the paternal obesogenic diets were associated overall with greater retroperitoneal fat (%BW) in the dams. *Post hoc* testing showed matOB‐patOB was significantly higher than matCON‐patCON and matCON‐patOB (Table 3).

### 3.4 Litter characteristics and body weights and lengths at birth (PN1)

There were no differences in litter sizes or male‐to‐female pup ratios across the four dietary groups (Table 4).

**TABLE 4:** Early‐life growth and metabolic outcomes in offspring from control and obesogenic parental diet combinations.

	matCON‐patCON	matCON‐patOB	matOB‐patCON	matOB‐ patOB	Maternal diet effect	Paternal diet effect	Parental diet interaction
**Litter characteristics**							
Number of litters	15	16	18	15			
Litter size	12.7 ± 1.4	12.4 ± 2.1	12.5 ± 1.5	11.9 ± 2.8	0.552	0.402	0.788
Sex ratio, % male per litter	49.4 ± 12.0	54.7 ± 8.9	47.9 ± 13.2	53.7 ± 15.5	0.684	0.0843	0.929
Sex ratio, % female per litter	50.6 ± 12.0	45.3 ± 8.9	52.1 ± 13.2	46.3 ± 15.5	0.684	0.0843	0.929
**Male offspring**							
PN1 weight, g	6.98 ± 0.78^a^	6.99 ± 0.69^a^	6.38 ± 0.94^b^	6.00 ± 0.90^c^	**<0.001**	**0.0314**	**0.0230**
PN1 length, mm	45.4 ± 3.1^a^	46.0 ± 3.2^a^	45.3 ± 0.42^a^	43.1 ± 0.34^b^	**<0.001**	**0.0353**	**<0.001**
PN21 weight, g	53.9 ± 5.0^a^	56.1 ± 4.5^a,b^	58.3 ± 4.8^b^	57.9 ± 7.1^b^	<**0.001**	0.205	0.0665
PN21 liver, %BW	3.72 ± 0.41	3.76 ± 0.30	4.00 ± 0.21	3.81 ± 0.49	0.0955	0.429	0.247
PN21 retroperitoneal fat, %BW	0.20 ± 0.05^a^	0.23 ± 0.08^a^	0.47 ± 0.08^b^	0.46 ± 0.13^b^	**<0.001**	0.747	0.405
PN21 leptin, ng/mL	5.01 ± 0.97^a^	5.11 ± 0.67^a,b^	8.85 ± 2.51^b^	7.73 ± 1.99^b^	**<0.001**	0.278	0.195
**Female offspring**							
PN1 weight, g	6.42 ± 0.72^a^	6.59 ± 0.68^a^	5.83 ± 0.80^b^	5.72 ± 0.87^b^	**<0.001**	0.692	0.0776
PN1 length, mm	44.3 ± 3.4^a^	44.0 ± 4.0^a^	43.5 ± 3.8^a^	42.0 ± 3.3^b^	**<0.001**	**0.0148**	0.0968
PN21 weight, g	52.4 ± 5.2^a^	55.4 ± 4.2^b^	56.8 ± 5.1^b^	57.4 ± 6.1^b^	**<0.001**	**0.0143**	0.0867
PN21 liver, %BW	3.67 ± 0.22	3.85 ± 0.39	3.99 ± 0.56	4.15 ± 0.48	**0.0152**	0.171	0.925
PN21 retroperitoneal fat, %BW	0.17 ± 0.08^a^	0.17 ± 0.05^a^	0.40 ± 0.11^b^	0.37 ± 0.06^b^	**<0.001**	0.505	0.460
PN21 leptin, ng/mL	5.16 ± 1.02^a^	5.01 ± 0.93^a^	8.63 ± 2.15^b^	7.09 ± 1.85^b^	**<0.001**	0.0620	0.120

*Note*: Birth size (PN1 weight and length: *n =* 92–118), weaning weight (PN21: *n =* 35–68) and organ weights, adiposity and plasma leptin levels at PN21 (*n =* 7–16) in male and female offspring across four parental diet groups. Data are presented as the mean ± SD. Groups that do not share a superscript letter in common are significantly different (*P* < 0.05; shown in bold) by parametric two‐way ANOVA, with Tukey's multiple comparison test.

Abbreviations: %BW, percentage of body weight; matCON‐patCON is the group where the mother and father consumed a control diet; matCON‐patOB, the mother consumed a control diet and the father an obesogenic diet; matOB‐patCON, the mother consumed an obesogenic diet and the father a control diet; matOB‐patOB, both parents consumed an obesogenic diet.

In male offspring, there were overall effects of maternal diet, paternal diet and parental diet interaction for birth weights and NA lengths at PN1. For birth weights, *post hoc* testing showed that matOB diet reduced weights, and these were significantly reduced further in the matOB‐patOB group (Table 4; Figure 6). For birth lengths, *post hoc* tests showed that the matOB‐patOB group had reduced body lengths compared with all other groups.

In female offspring, there was a significant overall maternal diet effect on reducing birth weights (Table 4), with an overall trend towards a parental diet interaction further reducing birth weights (*P* = 0.078). *Post hoc* testing showed that birth weights in the matOB‐patCON and matOB‐patOB groups were significantly reduced compared with the other groups (Table 4). For female NA lengths, there was an overall maternal and paternal diet effect. *Post hoc* testing showed that NA length was significantly reduced in the matOB‐patOB group compared with all other groups.

### 3.5 Neonatal mortality and survival until weaning (PN21)

**FIGURE 5:**
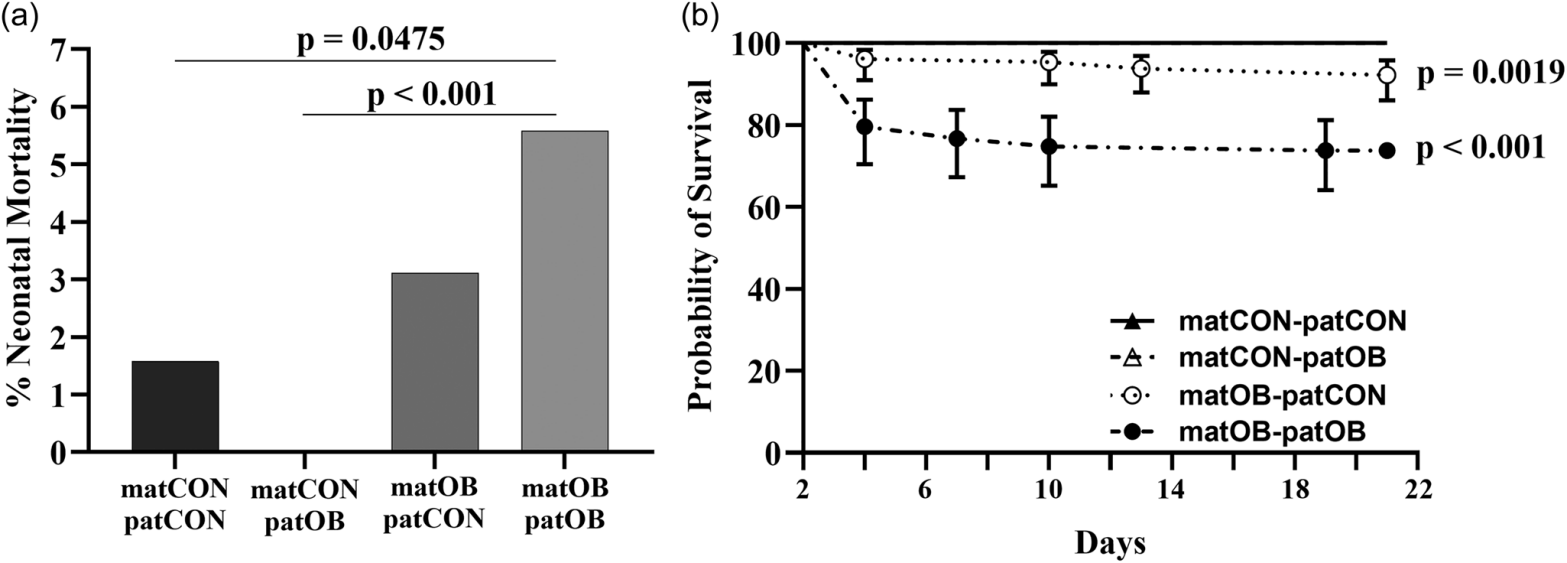
(a) Neonatal mortality in offspring across the four parental diet combinations. Sample sizes: matCON‐patCON (*n =* 190), matCON‐patOB (*n =* 198), matOB‐patCON (*n =* 225) and matOB‐patOB (*n =* 179). (b) Postnatal survival to weaning in the same offspring groups. Sample sizes: matCON‐patCON (*n =* 120), matCON‐patOB (*n =* 128), matOB‐patCON (*n =* 129) and matOB‐patOB (*n =* 103). Note that matCON‐patCON and matCON‐patOB groups are not plotted in (b), because there were no deaths between PN2 and weaning in these groups.

Neonatal mortality at PN1 was significantly increased in the matOB‐patOB group compared with matCON‐patCON and matCON‐patOB groups (Figure 5a). Offspring survival from PN2 until weaning (PN21) was significantly reduced in offspring from the matOB‐patCON group and significantly worsened further in offspring from the matOB‐patOB group (Figure 5b).

### 3.6 Body growth from birth until weaning

**FIGURE 6:**
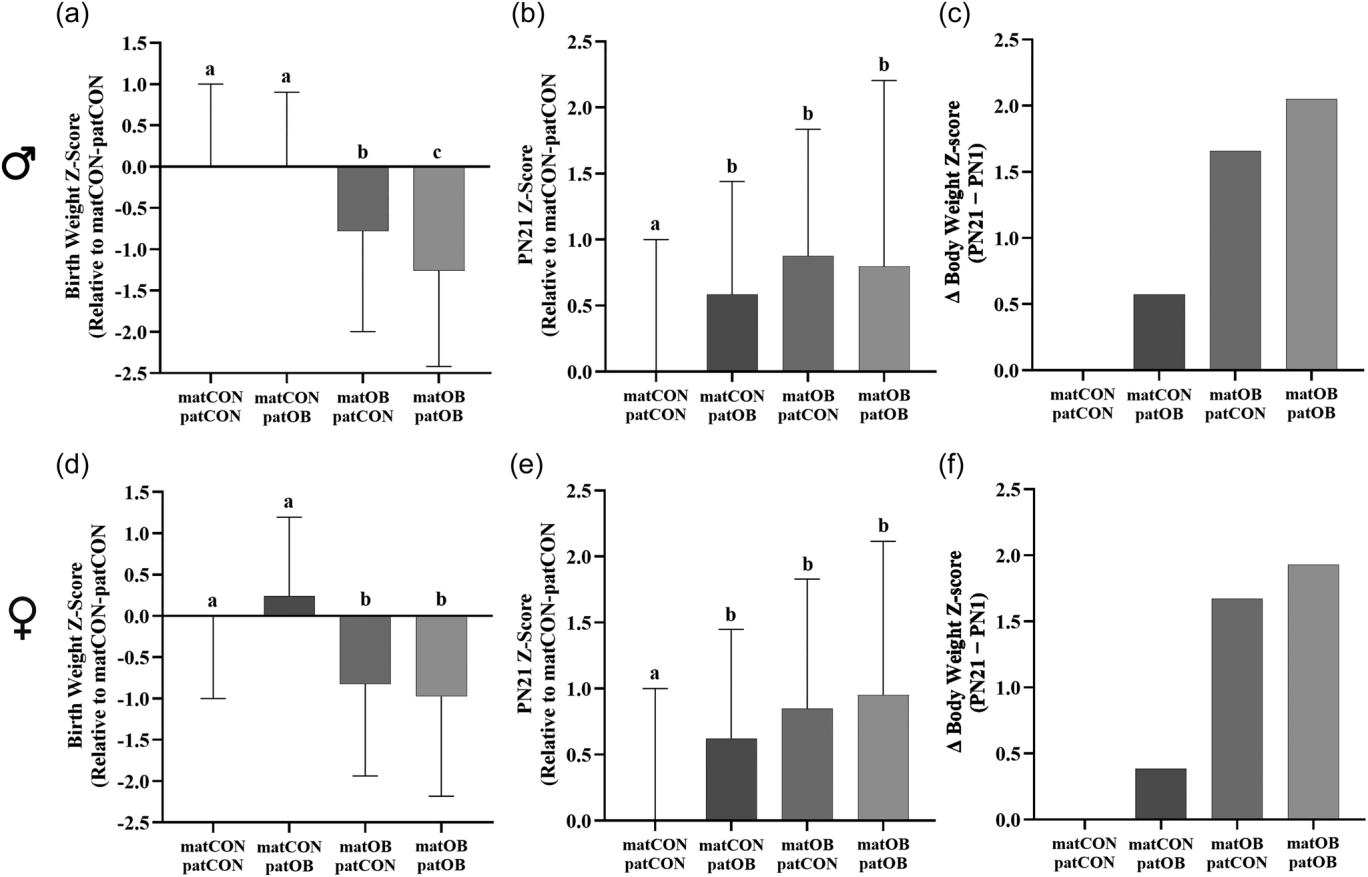
Body weight at birth and at weaning in male (a–c) and female (d–f) offspring from PN1 to PN21. (a, d) PN1 body weight *z*‐scores. (b, e) PN21 *z*‐scores. (c, f) Change in *z*‐score (Δ*z* = PN21 − PN1). Sample sizes ranged from *n =* 179 to 225. Data are presented as the mean ± SD; for (a), (b), (d) and (e), SD values are shown, whereas for (c) and (f), SD and significance could not be calculated. Groups that do not share a letter in common are significantly different (*P* < 0.05) by parametric two‐way ANOVA, with Tukey's multiple comparison test.

There was an overall maternal diet effect on male weaning (PN21) weights. *Post hoc* testing showed that weaning weights were increased in the matOB‐patCON and matOB‐patOB groups compared with matCON‐patCON, whereas no differences were observed between the matCON‐patOB group and other groups (Table 4; Figure 6). In female offspring, there was an overall maternal and paternal diet effect on weaning weights. *Post hoc* testing showed that PN21 weights were increased in all OB groups compared with matCON‐patCON. The rate of catch‐up growth, quantified as change in body weight *z*‐score from PN1 to PN21 (Δ*z* = PN21*z* − PN1*z*), was increased in all OB diet groups compared with matCON‐patCON (Figure 6).

### 3.7 Tissue weights and plasma leptin concentrations at weaning

In male offspring, there were no significant differences in relative liver weights (%BW) across any of the dietary groups (Table 4). For retroperitoneal fat (%BW), there was an overall maternal diet effect. *Post hoc* testing showed that retroperitoneal fat mass was increased in the matOB‐patCON and matOB‐patOB groups compared with other groups. There was an overall maternal diet effect for plasma leptin concentrations. *Post hoc* testing showed that leptin concentrations were increased in the matOB‐patCON and matOB‐patOB groups compared with the matCON‐patCON group.

In female offspring, there was an overall maternal diet effect increasing relative liver weights (%BW). However, *post hoc* testing showed no differences across the dietary groups (Table 4). For retroperitoneal fat mass and plasma leptin concentrations, there were overall maternal diet effects and a trend towards an overall paternal diet effect on reducing plasma leptin concentrations (*P* = 0.0620). *Post hoc* testing showed that retroperitoneal fat mass and plasma leptin was increased in the matOB‐patCON and matOB‐patOB groups compared with the other groups.

## 4 DISCUSSION

We investigated the independent and combined effects of obesogenic maternal and paternal diets on mating success, pregnancy, and early‐life outcomes for the offspring. Maternal obesity reduced survival of offspring during lactation and led to a phenotype characterised by reduced birth weight and length, with subsequent more rapid early weight gain leading to greater weight, adiposity and plasma leptin concentration at 21 days of life. The independent effects of paternal obesity on the offspring were less marked, with greater PN21 weight solely in female offspring. However, when both parents consumed obesogenic diets, offspring had increased neonatal mortality, poorer early‐life survival, lower birth weight and length, with greater catch‐up growth to day 21. These findings highlight the synergistic negative effects of maternal and paternal unhealthy diet on offspring early‐life outcomes.

The high‐fat, high‐sucrose diet was used because it shares features with unhealthy western diets characterised by high fat and high sugar content and it is known to induce obesity in rats (Ahmed et al., 2019; Kobi et al., 2023; Rawat et al., 2025; Zhou et al., 2014). As expected, it induced obesity with greater body weight and fat mass, with metabolic impairment in both sexes, in addition to a reduction in lean body mass.

In this study, paternal exposure to an obesogenic diet was associated with impaired fertility, as evidenced by a substantial reduction in the proportion of successful pregnancies following a mating attempt. We assessed the initial success of mating by examining for sperm in vaginal lavage fluid the next day and found a substantial reduction in positive lavage in the group where both parents consumed an obesogenic diet. This accounted almost entirely for the reduction in successful pregnancies in this group. In contrast, when only the male consumed the obesogenic diet, there was no impact on the rate of initial mating success, yet the rate of successful pregnancy was also reduced. This reduction of success in male‐only obesity might be attributable to alterations in sperm function, motility and fertilization capacity reported by other groups (Bakos et al., 2011; Ferramosca et al., 2016). However, in the context of combined maternal and paternal obesogenic diets, we speculate that there was a reduction in the willingness of the male to mate and/or the receptivity of the female, possibly through altered pheromones or behavioural cues.

The effect of the maternal obesogenic diet on dams was generally unsurprising, with greater caloric intake, greater post‐partum weight and greater liver and body fat at the end of lactation. Interestingly, there was a lesser reduction in weight after delivery in the obesogenic diet groups, which might represent the lower body weight of the pups, but could also represent lower placental weights. Perhaps surprisingly, the paternal obesogenic diet was also associated with changes in the dams. When the father had consumed the obesogenic diet, the dams had reduced gestational weight gain and greater retroperitoneal body fat at the end of lactation. This difference in gestational weight gain might relate to differences in fetal growth and the placentae, but the effect on maternal body fat suggests alteration in the metabolism of the dam. To our knowledge, an effect of paternal diet or health on the body composition of the mother has not been reported. The mechanism is unknown but could relate to differences in placental function. For example, paternal inactivation of the imprinted *Phlda2* gene in mice leads to greater expansion of the spongiotrophoblast, which secretes placental lactogen (Creeth et al., 2018). Thus, it is plausible that the paternal obesogenic diet leads to altered secretion of placental hormones that modulate metabolism, such as placental lactogen and placental growth hormone, through an epigenetic mechanism (Newbern & Freemark, 2011).

Neonatal mortality was highest in offspring from the combined maternal–paternal obesogenic diet group (matOB‐patOB), whereas there was no neonatal loss associated with paternal obesity alone. Survival from birth to weaning was reduced in offspring exposed to maternal obesity, with the most pronounced effects observed when both parents were fed an obesogenic diet. Furthermore, it is possible that the effect of the parental diets on survival was underestimated. On PN2, when animals were culled, less vigorous pups were preferentially selected for cull, which might have included pups that would have died if they were not removed. Although the underlying mechanisms driving this increased mortality are unclear, the groups with increased mortality had pups born with lower body weight and length, implying differences in fetal growth. Such differences are likely to be related to impaired placental function (Reynolds et al., 2015), which could have affected the fitness of the pups after delivery. It is also plausible that there were differences in maternal care or lactation (e.g., milk volume or composition) on the first day associated with a maternal high‐fat diet (Connor et al., 2012). The compounding effects of the paternal obesogenic diet could also have affected the metabolism or behaviours of the mother more directly, for example, through an effect on placental function, including hormone secretion (Fleming, 2018). These possibilities would need to be addressed in a future study.

Our findings demonstrate that maternal obesity reduced birth weight in male and female offspring, with a greater reduction in male offspring when the father also consumed an obesogenic diet. Reductions in body length were seen only when both parents consumed the obesogenic diet. This indicates poorer fetal growth and nutrition and is likely to represent a negative impact on placental function that is more severe in the setting of combined parental obesity. Poorer placental function has been demonstrated in models of maternal obesity (Reynolds et al., 2015). Function of the placenta, which is a combined maternal and fetal organ, is potentially affected by maternal health and by maternal and paternal epigenetic inheritance (Basak et al., 2024; Bhadsavle & Golding, 2022), meaning that an interaction between the maternal and paternal diets is not surprising.

These early growth impairments associated with a maternal obesogenic diet were followed by more rapid weight gain from birth to weaning and greater adiposity and higher plasma leptin. The greatest Δweight *z*‐score (PN1–PN21) was seen in the group where both parents consumed an obesogenic diet. This early‐life growth pattern has previously been described as ‘catch‐up fat’, because fat tissue accumulates preferentially over lean mass and has been linked to early‐onset metabolic disorders and long‐term obesity risk (Dulloo et al., 2006). When induced by a maternal high‐fat diet, this phenotype is associated with adverse effects on development of metabolic tissues, including liver, adipose tissue, skeletal muscle and pancreas, in addition to altering expression of genes in the hypothalamus that modify appetite and energy expenditure (Harmancıoğlu & Kabaran, 2023). We speculate that there was an interaction between the maternal and paternal diets on the function of these tissues.

In this study, there were minimal effects of the paternal obesogenic diet alone on offspring or pregnancy outcomes. This fits with the obvious importance of maternal health on pregnancy and the offspring, because it is the mother who carries the pregnancy, provides early nutrition through lactation and cares for the offspring. In contrast, in the rat model, the father contributes only during the act of mating, through the provision of sperm and seminal fluid. However, there were important interactions between the paternal and maternal diets, whereby a paternal obesogenic diet was much more harmful to the offspring if the mother was also consuming a high‐fat diet. This highlights the importance of studying parental diets in combination in order that such interactions can be identified. Furthermore, it raises important questions about the mechanism for these interactions.

Increasing evidence supports a role for alterations in sperm epigenetics as a major pathway through which paternal health impacts offspring. This could be mediated through differences in DNA methylation, microRNA load or histone acetylation, all of which are altered in models of paternal obesity (Fullston et al., 2016). In particular, DNA methylation appears important, with paternal obesity or unhealthy diet being associated with hypomethylation of genes that regulate fetal growth and are associated with risk of obesity in offspring, including *IGF2* (Akhatova et al., 2025). The microRNA in sperm was once considered unimportant but might play a role in offspring disease risk, because injection of specific microRNAs into mouse eggs has been demonstrated to alter a variety of offspring outcomes (Dupont et al., 2019). Recently, paternal obesity has been shown to alter histone acetylation in sperm chromatin associated with genes involved in metabolism and development of the offspring and also placental function (Pepin et al., 2024). Future work should examine the interacting effects of maternal and paternal obesogenic diets on offspring and placental epigenetics, to gain a better understanding of the interactions we have described.

The main strengths of this study are its well‐powered and balanced design that enabled the independent and synergistic effects of maternal and paternal obesogenic diets to be examined. Although these results are novel, they are descriptive in nature. This study was not designed to elucidate mechanism. There is a need for further studies to link specific changes in sperm epigenetics to the phenotype and epigenetic profiles of the offspring, to assess the mechanisms around early survival and the mechanism linking the paternal obesogenic diet to increased body fat in dams.

## 5 CONCLUSION

Parental exposure to obesogenic diets impairs reproductive and early developmental outcomes. Paternal obesity alone reduced mating success, indicating compromised male fertility. Maternal obesity lowered birth weights in both sexes, and paternal obesity further reduced birth weight in male offspring when combined with maternal obesity. Neonatal mortality increased when both parents were obese, reflecting additive effects on early survival.

Postnatal growth patterns were also altered. Maternal obesity promoted early catch‐up growth and adiposity in both sexes. Paternal obesity accelerated early weight gain in females. Combined parental obesity intensified these effects, leading to increased fat accumulation and rapid growth in offspring. At weaning, plasma leptin concentrations were significantly elevated in offspring exposed to maternal obesity, reflecting their increased fat mass.

These findings highlight the distinct and combined impacts of maternal and paternal nutritional status on reproductive success, fetal growth, neonatal viability and early‐life metabolic outcomes. To address the global obesity epidemic, there might be a need to optimise the diet and health of both the mother and the father prior to conception.

## AUTHOR CONTRIBUTIONS

Benjamin B. Albert, Mark H. Vickers and David S. Musson conceived the research. Benjamin B. Albert, Mark H. Vickers, David S. Musson and Anna P. Ponnampalam designed this study. Khurram Jahangir Toor, Mehwish Abbasi, Swetha Muralidharan, Elwyn C. Firth, Anna P. Ponnampalam, David S. Musson, Elisa Weiss, Mark H. Vickers and Benjamin B. Albert carried out this study. Khurram Jahangir Toor, Mark H. Vickers and Benjamin B. Albert statistically analysed this study. Khurram Jahangir Toor, Mark H. Vickers, Anna P. Ponnampalam and Benjamin B. Albert interpreted data. Benjamin B. Albert led the funding acquisition. Khurram Jahangir Toor, Benjamin B. Albert, Mark H. Vickers and Anna P. Ponnampalam prepared the original draft. All authors contributed to review and editing of the manuscript, approved the final version of the manuscript and agree to be accountable for all aspects of the work in ensuring that questions related to the accuracy or integrity of any part of the work are appropriately investigated and resolved. All persons designated as authors qualify for authorship, and all those who qualify for authorship are listed.

## CONFLICT OF INTEREST

None declared.

## Data Availability

Data are available on request to the corresponding author.
